# Prevalence and risk factors for carbapenem-resistant Enterobacterales colonization in hospitalized children in a provincial hospital in Vietnam

**DOI:** 10.3389/fcimb.2026.1770367

**Published:** 2026-02-20

**Authors:** Van Anh Thi Dinh, Nhung Nguyen Thi Trang, Ngoc Duc Vo, Ngoc Hoang Thi Bich, Dien M. Tran, Pham Duc Phuc, Son Luong Duc, Do Manh Dung, Flavie Goutard, Thirumalaisamy P. Velavan, Dennis Nurjadi, Yaovi M. G. Hounmanou, Bent Jörgensen, Le Huu Song, Truong Nhat My, Etienne Loire, Åse Östholm, Lennart E. Nilsson, Phuc H. Phan, Anders Dalsgaard, Mattias Larsson, I-CRECT Consortium, Håkan Hanberger, Bent Jörgensen, Åse Östholm, Lennart E Nilsson, Mattias Larsson, Linus Olson, Thirumalaisamy P. Velavan, Song H. Le, Alexa Purgreth, Mai Thi Pham, Flavie Goutard, Etienne Loire, Y.M. Gildas Hounmanou, Anders Dalsgaard, Phuc D. Pham, Hanh T.T. Tran, Nhung T. T. Nguyen, Dien M. Tran, Phuc H. Phan, Ngoc T.B Hoang, Van Anh Thi Dinh, Ngai K. Le, Linus Olson, Håkan Hanberger

**Affiliations:** 1 Department of Infection Control, Vietnam National Children’s Hospital, Hanoi, Vietnam; 2 The Training and Research Institute of Child Health, Hanoi, Vietnam; 3 Hanoi University of Public Health, Hanoi, Vietnam; 4 Department of Microbiology, Vietnam National Children’s Hospital, Hanoi, Vietnam; 5 Vietnam National Children’s Hospital, Hanoi, Vietnam; 6 University of Medicine and Pharmacy - Vietnam National University, Hanoi, Vietnam; 7 Thai Binh Paediatric Hospital, Hưng Yên, Vietnam; 8 The French Agricultural Research Centre for International Development (CIRAD), Montpellier, France; 9 Institute of Tropical Medicine, Universitätsklinikum Tübingen, Tübingen, Germany; 10 Vietnamese German Center for Medical Research (VG-CARE), Hanoi, Vietnam; 11 Faculty of Medicine, Duy Tan University, Da Nang, Vietnam; 12 Institute of Medical Microbiology, University of Lübeck and University Hospital Schleswig-Holstein, Lübeck, Germany; 13 Faculty of Health and Medical Sciences, University of Copenhagen, Copenhagen, Denmark; 14 Division of Infectious Diseases, Department of Biomedical and Clinical Sciences, Faculty of Medicine, Linköping University, Linköping, Sweden; 15 Training and Research Academic Collaboration (TRAC) – Sweden – Vietnam, Hanoi, Sweden; 16 School of Global Studies, University of Gothenburg, Gothenburg, Sweden; 17 108 Military Central Hospital, Hanoi, Vietnam; 18 International Centre For Antimicrobial Resistance Solutions, Copenhagen, Denmark; 19 Department of Global Public Health, Karolinska Institutet, Stockholm, Sweden; 20 Department of Women’s and Children’s Health, Karolinska Institutet, Stockholm, Sweden

**Keywords:** Carbapenem-Resistant Enterobacterales, CRE colonization, hospital acquired infection, pediatric hospital, point prevalence survey, Vietnam

## Abstract

**Background and aims:**

Carbapenem-resistant Enterobacterales (CRE) colonization is an important prerequisite to hospital-acquired infections (HAIs) caused by CRE and increased mortality. This study assessed the prevalence of, and risk factors for, CRE colonization among children admitted to a provincial pediatric hospital in a high–antimicrobial-resistance setting in Vietnam.

**Methods:**

A point prevalence survey was conducted on 15 August 2022 at a provincial pediatric hospital in the Red River Delta. Rectal swabs were collected from 376 hospitalized children after informed consent. Samples were inoculated on chromogenic selective agar for CRE detection, and bacterial identification and antimicrobial susceptibility testing were performed using the VITEK^®^ 2 system. Clinical and demographic data were obtained from structured questionnaires and medical records. Logistic regression analyses were used to identify risk factors associated with CRE colonization.

**Results:**

CRE colonization was detected in 28.2% of hospitalized children. Colonization was more common among patients screened >48 hours after admission (30.8%) and hospitalization >48 hours was associated with a 2.27-fold increased risk of colonization (p = 0.026) compared with screening earlier. Prevalence increased with length of stay, reaching 40.8% among those hospitalized ≥7 days. Children ≤6 years had a colonization rate of 29.7%. The highest ward-level prevalence occurred in the Intensive Care Unit (77.8%), whereas the lowest rates were observed in trauma and nutrition wards (9.5%). *Escherichia coli* (40.6%), *Klebsiella pneumoniae* (34.4%), and *Enterobacter cloacae* (16.4%) were the predominant CRE species. Most isolates were resistant to 4–10 antimicrobial classes. CRE colonization was also significantly associated with sepsis at the time of assessment.

**Conclusions:**

CRE colonization was highly prevalent among pediatric inpatients in this provincial Vietnamese hospital, particularly among young children, those hospitalized for prolonged periods, and patients in intensive care or neonatal units. These findings underscore the urgent need to strengthen infection prevention and control (IPC) strategies and to implement targeted screening approaches to assess the effect on CRE transmission of improvement of IPC.

## Highlights

- Carbapenem-resistant Enterobacterales (CRE) pose a major public health threat due to limited treatment options and their association with prolonged hospitalization and poor clinical outcomes.
- A point prevalence survey in a provincial pediatric hospital in Vietnam found a 28.2% prevalence of CRE colonization among hospitalized children.
- Colonization was highest among neonates and children admitted to intensive care units.
- Key risk factors for CRE colonization included hospitalization for >48 hours and age ≤6 years, and admission to ICU or neonatal units.
- The findings underscore the urgent need to strengthen infection prevention and control measures in low- and middle-income settings to reduce in-hospital CRE transmission and potential household dissemination after discharge.

## Introduction

Carbapenem-resistant Enterobacterales (CRE) are recognized as among the most critical priority antimicrobial resistant pathogens and represent a major global threat to human health and antimicrobial resistance. Their emergence is particularly concerning in inpatient settings, where vulnerable patients and high antimicrobial use facilitate selection and transmission, underscoring the need for robust infection prevention and control (IPC) strategies (World Health, O, 2017). CRE prevalence varies considerably across healthcare environments, ranging from 1% to 30.4% in long-term care facilities in the United States and from 13% to 22.7% across Asian hospitals (Chen et al., 2021). In a neonatal intensive care unit (NICU) in Shanghai, China, the CRE colonization rate reached 11.7% during 2017–2018 (Yin et al., 2021).

In Vietnam, carbapenems have been used clinically since 2008 (Nguyen et al., 2013), and increasing reports of carbapenem-resistant organisms—including strains producing NDM-1—reflect the growing burden of resistance nationwide (Hoang et al., 2013; Tran et al., 2015). A multicenter survey involving 12 hospitals showed that 52% of screened patients were colonized with CRE, with prevalence rising from 13% at admission to 89% by hospital day 15; in one provincial pediatric hospital, carriage increased from 29% to 53% over a 16-month period (Tran et al., 2019). These data highlight both high background colonization pressure and rapid in-hospital acquisition.

A national survey of Vietnamese intensive care units reported HAIs in 30% of approximately 5,000 admitted patients (Le et al., 2016), and mortality among neonates with culture-confirmed HAIs caused by carbapenem-resistant Gram-negative bacteria exceeded 50% (Peters et al., 2019). CRE colonization is a prerequisite to subsequent invasive infection. Clearance of colonization is protracted, with median persistence of roughly one year, and prolonged carriage was associated with therapeutic antimicrobial exposure (Kramer et al., 2013).

High CRE colonization rates at discharge raise concerns about onward dissemination to household members, the community, and the environment. Such transmission may further complicate the management of community-onset infections, including urinary tract infections that may fail standard empirical therapy, even when treated with intravenous agents such as amikacin. Evidence from a longitudinal study of extended-spectrum β-lactamase (ESBL)-producing Enterobacterales demonstrated high within-household transmission probabilities - 67% from index patient to household contact and 37% in the reverse direction - and prolonged carriage associated with antibiotic exposure (Haverkate et al., 2017). Although focused on ESBL organisms with resistance to third generation cephalosporins, these findings underscore the potential for similar dynamics for CRE.

Early and accurate detection of CRE colonization is critical for IPC, outbreak containment, and guiding clinical management, particularly in high-risk units such as NICUs and ICUs (Zaidah et al., 2017). This study aimed to determine the prevalence and risk factors for CRE colonization among children hospitalized in a provincial pediatric hospital in Vietnam. The findings are intended to inform the development and implementation of targeted strategies to mitigate CRE transmission within healthcare facilities and reduce dissemination to the community.

## Methods

### Setting

The study was conducted at a provincial pediatric hospital in the Red River Delta, a densely populated region of northern Vietnam (1,185 inhabitants/km² in 2022) (United Nations and General Statistics Office Vietnam, 2024). The hospital provides comprehensive pediatric care, with 600 inpatient beds and 25,620 admissions in 2022, and hosts an on-site microbiology laboratory capable of performing culture, identification, and antimicrobial susceptibility testing. The mean inpatient stay was 7.4 days, with substantially longer durations in the Neonatal, Intensive Care, and Respiratory Departments (22.9, 10.6, and 7.9 days, respectively). In 2022, 71 hospital-acquired infections (HAIs) were recorded, highlighting the importance of strengthening infection prevention and control (IPC) measures (**Appendix 3**). Given previously documented high CRE colonization rates and the hospital’s commitment to IPC improvement, the facility was selected as a study site within the randomized controlled trial entitled *Interventions to Reduce CRE Colonization and Transmission Across Hospitals, Households, Communities, and Domesticated Animals*.

### Study population

All inpatients <18 years of age admitted to any of the 13 clinical departments were eligible for inclusion. A total of 376 caregivers provided written informed consent for participation and specimen collection.

### Study design

A cross-sectional point prevalence survey study was conducted on August 15, 2022.

### Data collection

Data were collected using a structured questionnaire with coded identifiers to ensure confidentiality. Informed consent was obtained from caregivers prior to enrolment. Trained clinicians conducted interviews and extracted clinical information from medical records.

Collected data included: 1) Household characteristics: address, number of household members, animal husbandry practices, household conditions, water source, and reported antimicrobial use; 2) Clinical information: age, sex, diagnosis at admission, symptoms, admission and discharge dates, hospital department, source of referral, prior antimicrobial exposure, and previous hospitalizations; 3) Microbiological samples: faucal specimens obtained using rectal swabs.

### Microbiological methods

Rectal swabs were collected using sterile cotton swabs and delivered to the hospital microbiology laboratory within two hours. Samples were inoculated onto MELAB Chromogenic Carba agar (Lavitec, Vietnam), a locally manufactured equivalent of CHROMagar™ mSuperCARBA™, for CRE screening (Lavitec, [[NoYear]]) Plates were subsequently transported within two hours to the Vietnam National Children’s Hospital for incubation at 35 ± 2°C for 18–24 hours.

Bacterial isolates were identified to species level and tested for antimicrobial susceptibility using the VITEK^®^ 2 XL system (bioMérieux, France). Susceptibility testing included agents across 11 antimicrobial classes: aminoglycosides; fluoroquinolones; antipseudomonal penicillins with β-lactamase inhibitors; carbapenems; non-extended-spectrum cephalosporins; first- and second-generation cephalosporins; cephamycins; third- and fourth-generation cephalosporins; penicillins; penicillins with β-lactamase inhibitors; phosphonic acids; and folate-pathway inhibitors. AST interpretation followed current Clinical and Laboratory Standards Institute (CLSI) breakpoints (Clinical and Laboratory Standards Institute, C, 2021). All isolates were stored at –80°C for future analyses.

Multidrug resistance (MDR) was defined according to Magiorakos (Magiorakos et al., 2012): non-susceptibility to ≥1 agent in ≥3 antimicrobial classes.

### Statistical analysis

Data analysis was conducted using R Statistical Software (v4.5.1; R Core Team 2025). Descriptive statistics summarized demographic and clinical characteristics. Visualizations were produced with the *ggplot*2 package (v4.0.0).

Multivariable logistic regression was used to identify risk factors for CRE colonization. Covariates included age, sex, diagnosis at the time of the survey, prior antimicrobial exposure, residential address, time to detection after hospital administration, and previous hospitalization. Associations were expressed as crude odds ratios (ORs) from univariate analyses and adjusted ORs from multivariable models. Statistical significance was defined as *p < 0.05*.

### Quality control

Standardized training was provided to all data collectors and laboratory personnel to ensure methodological consistency and accuracy. Laboratory procedures at the Vietnam National Children’s Hospital microbiology department adhere to ISO 15189 standards.

### Ethical considerations

Ethical approval for this study and the overarching I-CRECT project (“*Intervention to Decrease CRE Colonization and Transmission Between Hospitals, Households, Communities and Domesticated Animals*”) was obtained from Ministry of Health, Vietnam, and performed after ethical approval from Ethical Review Board of Vietnam Children’s Hospital (approval code VNCH-TRICH-2022–87 dated 30th September, 2022 issued by Vietnam Ministry of Health and Ethical Review Board of Hanoi University of Public Health (approval code 022-350/DD-YTCC, 25 July, 2022).

## Results

Among the 376 hospitalized children included in the survey, the overall prevalence of CRE colonization was 28.2% (**Table 1**). Children ≤6 years accounted for most of the study population (344/376, 91.5%) and had a higher prevalence of colonization (29.7%) compared with those >6 years (12.5%). Similarly, children ≤3 years exhibited a CRE prevalence of 30.3%. CRE colonization did not differ markedly by sex, with colonization observed in 28.9% of males and 26.7% of females.

**Table 1:** Demographic and clinical characteristics of hospitalized children and distribution of carbapenem-resistant Enterobacterales (CRE) colonization in a provincial pediatric hospital, Red River Delta, Vietnam, 2022.

Characteristic	Number of patients with negative CRE	Number of patients with positive CRE	Total
Age group			
≤ 6 years	242 (70.3%)	102 (29.7%)	344
> 6 years	28 (87.5%)	4 (12.5%)	32
≤ 3 years	205 (69.7%)	89 (30.3%)	294
Gender			
Male	170 (71.1%)	69 (28.9%)	239
Female	99 (73.3%)	36 (26.7%)	135
Missing data	1 (50%)	1 (50%)	2
Referral source			
Other hospital	0 (0%)	3 (100%)	3
Other department	11 (100%)	0 (0%)	11
Lower-level medical facility	20 (80%)	5 (20%)	25
Direct from home	220 (70.1%)	94 (29.9%)	314
No data	19 (82.6%)	4 (17.4%)	23
**Delivery mode (neonatal only)**	22 (61.1%)	14 (38.9%)	36
Normal delivery	14 (63.6%)	8 (36.4%)	22
Caesarean section	6 (50%)	6 (50%)	12
Missing data	2 (100%)	0 (0%)	2
Timing of carbapenem screening			
≤ 48 hours after admission	58 (82.9%)	12 (17.1%)	70
> 48 hours after admission	211 (69.2%)	94 (30.8%)	305
Missing data	1 (100%)	0 (0%)	1
Length of stay ≥ 7 days	74 (59.2%)	51 (40.8%)	125
**Total**	**270 (71.8%)**	**106 (28.2%)**	**376**

Most patients had been admitted directly from home (314/376), and this group showed a colonization prevalence of 29.9%. In contrast, children transferred from other hospitals or lower-level medical facilities had smaller sample sizes but also demonstrated notable colonization rates. (please see **Table 1**).

CRE colonization was 17.1% of among children screened within 48 hours of admission, and 30.8% after 48 hours. The highest prevalence was observed in patients with stays ≥7 days, 40.8% of whom were colonized. In the neonatal subgroup, CRE prevalence reached 38.9%. Overall, these findings indicate substantial colonization pressure across age groups and admission pathways, with markedly increased prevalence associated with prolonged hospitalization.

The prevalence of CRE colonization varied markedly across hospital departments (**Table 2**). The highest prevalence was observed in the Intensive Care Unit, where 77.8% of patients were colonized. High rates of colonization rates were also seen in the 3 Specialities Department (43.7%), the Neonatal Department (38.9%), and the Respiratory and Infectious Diseases Departments (both 34.4%). In contrast, substantially lower rates were found in the Trauma (9.5%), Nutrition (10.0%), and Emergency (13.3%) Departments. Intermediate prevalence levels were reported in Gastroenterology (24.1%), Cardiology (23.5%), and Nephrology–Urology (16.3%). These findings indicate substantial differences in CRE burden across clinical areas, with intensive care and neonatal services representing the highest-risk settings.

**Table 2:** Prevalence of carbapenem-resistant Enterobacterales (CRE) colonization among hospitalized children by clinical department.

Department	Neg	Pos	Total
ICU	4 (22.2%)	14 (77.8%)	18
3 specialities (Ophthalmology; Odonto-Stomatology and Maxillofacial Surgery; Otorhinolaryngology)	9 (56.3%)	7 (43.7%)	16
Neonatal	22 (61.1%)	14 (38.9%)	36
Respiratory	42 (65.6%)	22 (34.4%)	64
Infectious Diseases	21 (65.6%)	11 (34.4%)	32
Traditional medicine	9 (69.2%)	4 (30.8%)	13
Gastroenterology	22 (75.9%)	7 (24.1%)	29
Cardiology	13 (76.5%)	4 (23.5%)	17
Nephrology - Urology	36 (83.7%)	7 (16.3%)	43
Pediatrics	51 (82.3%)	11 (17.7%)	62
Emergency	13 (86.7%)	2 (13.3%)	15
Trauma	19 (90.5%)	2 (9.5%)	21
Nutrition	9 (90%)	1 (10%)	10
**Total**	**270 (71.8%)**	**106 (28.2%)**	**376**

A total of 128 CRE isolates were recovered from 106 colonized patients, corresponding to an average of 1.2 isolates per patient (**Table 3**). *Escherichia coli* was the most frequently identified species (40.6%), followed by *K. pneumoniae* (34.4%) and *E. cloacae* (16.4%). Species distribution varied across departments. In the ICU, *E. coli* and *K. pneumoniae* predominated, together accounting for 75% of isolates, while *Pseudomonas* spp. constituted an additional 15%. In the Neonatal Department, *K. pneumoniae* and *E. cloacae* were equally common (both 35.3%). Respiratory wards showed a similar dominance of *E. coli* and *K. pneumoniae* (each 42.9%). Less commonly isolated species included *K. oxytoca*, *Citrobacter freundii*, *Aeromonas sobria*, *Acinetobacter* spp., and *Brevundimonas diminuta*. These findings indicate that the CRE reservoir in the hospital is primarily composed of *E. coli* and *K. pneumoniae*, with notable departmental variation suggesting multiple ecological niches for CRE persistence and transmission.

**Table 3:** Number and species distribution of carbapenem-resistant Gram-negative isolates by department.

Department	CRE-positive patients (n)	Total isolate (n)	*E. coli (%)*	*Klebsiella pneumoniae (%)*	*Enterobacter cloacae (%)*	*K.oxytoca (%)*	*C. freundii (%)*	Other # (%)
Respiratory	22	28	12 (42.9%)	12 (42.9%)	2 (7.1%)			2 (7.1%) *Aeromonas sobria, Acinetobacter* sp
Neonatal	14	17	4 (23.5%)	6 (35.3%)	6 (35.3%)			1 (5.9%) *Acinetobacter* sp
ICU	14	20	7 (35%)	8 (40%)	2 (10%)			3 (15%) *Pseudomonas* sp
Infectious Diseases	11	12	4 (33.3%)	4 (33.3%)	4 (33.3%)			0 (0%)
General Internal Medicine	11	13	5 (38.5%)	5 (38.5%)	2 (15.4%)	1 (7.6%)		0 (0%)
Nephrology – Urology	7	9	4 (44.4%)	2 (22.2%)	1 (11.1%)			2 (22.2%) *Brevundimonas diminuta*
3 Specialities	7	7	3 (42.9%)	2 (28.5%)	1 (14.3%)		1 (14.3%)	
Gastroenterology	7	8	4 (50%)	0 (0%)	3 (37.5%)			1 (12.5%) *Aeromonas sobria*
Cardiology	4	4	4 (100%)	0 (0%)	0 (0%)			0 (0%)
Traditional medicine - Rehabilitation	4	4	2 (50%)	2 (50%)	0 (0%)			0 (0%)
Trauma	2	2	1 (50%)	1 (50%)	0 (0%)			0 (0%)
Emergency	2	3	2 (66.7%)	1 (33.3%)	0 (0%)			0 (0%)
Nutrition	1	1	0 (0%)	1 (100%)	0 (0%)			0 (0%)
**Total**	**106**	**128**	**52 (40.6%)**	**44 (34.4%)**	**21 (16.4%)**	**1** **(0.8%)**	**1** **(0.8%)**	**9** **(7.0%)**

Antimicrobial susceptibility patterns showed uniformly high resistance across CRE species (**Table 4**). All *E. coli*, *K. pneumoniae*, and *E. cloacae* isolates demonstrated 100% resistance to carbapenems and near-panresistance to 1st- and 2nd-generation cephalosporins, 3rd- and 4th-generation cephalosporins, cephamycins, and piperacillin–tazobactam. Fluoroquinolone resistance was also high, ranging from 90.1% in *E. coli* to 97.7% in *K. pneumoniae*. Aminoglycoside resistance was common, particularly among *K. pneumoniae* (84.1%). Fosfomycin resistance varied by species, from low levels in *E. coli* (3.8%) to high levels in *E. cloacae* (71.4%). Multidrug resistance (MDR), defined as combined resistance to 3rd-generation cephalosporins, fluoroquinolones, and aminoglycosides, was most prevalent in *K. pneumoniae* (79.5%) and *E. cloacae* (76.2%). Overall, the isolates exhibited extensive resistance across nearly all tested antimicrobial classes, leaving very limited therapeutic options.

**Table 4:** Antimicrobial resistance profiles of CRE species.

Antimicrobial group/agent	*Escherichia coli (n=52)*	*Klebsiella pneumoniae (n=44)*	*Enterobacter cloacae (n=21)*
Aminoglycoside (gentamicin/amikacin)	26 (50%)	37 (84.1%)	16 (76.2%)
Antipseudomonal penicillins + β-lactamase inhibitors (piperacillin-tazobactam)	50 (96.2%)	44 (100%)	21 (100%)
Carbapenems (ertapenem/meropenem)	52 (100%)	44 (100%)	21 (100%)
Non-extended spectrum cephalosporins; 1st and 2nd generation (cefuroxime)	52 (100%)	43 (97.7%)	21 (100%)
3^rd^-4th generation cephalosporin (cefotaxime/cefepime/ceftazidime)	50 (96.2%)	43 (97.7%)	21 (100%)
Cephamycins (Cefoxitin)	50 (96.2%)	43 (97.7%)	21 (100%)
Fluoroquinolones (ciprofloxacin)	47(90.1%)	43 (97.7%)	20 (95.2%)
Folate pathway inhibitors (trimethoprim-sulphamethoxazole)	41 (78.8%)	24 (54.5%)	13 (61.9%)
Penicillins (ampicillin)*	52 (100%)	Not tested	Not tested
Penicillins + β-lactamase inhibitors (amoxicillin-clavulanic acid)*	52 (100%)	44 (100%)	Not tested
Phosphonic acids (fosfomycin)	2 (3.8%)	12 (27.3%)	15 (71.4%)
MDR phenotype (combined resistance to 3rd generation cephalosporins, fluoroquinolones, and aminoglycosides)	23 (44.2%)	35 (79.5%)	16 (76.2%)

*Not tested for *K. pneumoniae* and *E. cloacae* where intrinsic resistance applies.

Multivariable analysis identified several significant risk factors for CRE colonization (**Table 5**). Children screened more than 48 hours after admission had more than twice the odds of being colonized compared with those screened within 48 hours (aOR 2.27, 95% CI 1.10–4.67, p = 0.026), indicating high in-hospital acquisition. Age ≤6 years was associated with higher colonization in crude analysis (cOR 2.93, p = 0.049), although this association did not remain statistically significant after adjustment. Sepsis at the time of the survey was strongly associated with CRE colonization, with affected children having more than sixfold increased odds to be CRE colonized compared with those with other diagnoses (aOR 6.54, 95% CI 1.55–27.64, p = 0.011). Unfortunately, data obtained did not allow us to determine whether patients had sepsis due to CRE or were septic due to other causes and colonized with CRE. Other diagnostic categories, including respiratory infections and diarrhea, did not show significant associations to CRE colonization. Overall, prolonged hospitalization and severe systemic illness emerged as the strongest predictors of CRE colonization in this cohort.

**Table 5:** Risk factors for CRE colonization (n= 374^a^).

Characteristic	n	cOR (95%CI)	p -value	aOR (95%CI)^+^	p-value
Age group					
> 6 years (reference)	32	1.00		1	
≤ 6 years	342	2.93 (1.00 - 8.58)	0.049^b^	2.7 (0.86 - 8.47)	0.090
Time from admission to CRE screening					
≤ 48 hours (reference)	69	1.00		1	
> 48 hours	305	2.35 (1.18 - 4.68)	0.015 ^b^	2.27 (1.1 - 4.67)	0.026 ^b^
Diagnosis at time of study					
Other diagnoses (reference)	90	1,00		1	
Lower respiratory tract infection	216	1.68 (0.94 - 3.01)	0.081	1.4 (0.74 - 2.64)	0.303
Upper respiratory tract infection	31	1.78 (0.72 - 4.41)	0.213	1.52 (0.58 - 3.97)	0.392
Sepsis	10	5.61 (1.44 - 21.9)	0.013 ^b^	6.54 (1.55 - 27.64)	0.011^b^
Diarrhea	27	0.47 (0.13 - 1.72)	0.252	0.48 (0.13 - 1.85)	0.289

a Two patients with missing data were excluded from the regression models.

b Statistically significant at p < 0.05.

The multivariable analysis was adjusted for gender, referral from hospital, prior antibiotic exposure and residential address.

## Discussion

In this point prevalence survey (PPS), we found a CRE colonization rate of 28.2% among hospitalized children in a provincial pediatric hospital. Colonization was particularly common in high-risk units, with prevalence of 77.8% in the ICU, 43.7% in the 3 Specialities Department, 38.9% in the Neonatal Department, and 34.4% in the Respiratory Department, whereas the lowest rates were observed in the Nutrition (10%) and Trauma (9.5%) Departments. These findings are comparable to, and in some instances higher than, earlier reports from Viet Nam and other settings. At the Vietnam National Children’s Hospital, a cross-sectional survey conducted from March to June 2018 reported a CRE carriage rate of 35% among ICU inpatients (Garpvall et al., 2021). Most of those patients had previously been treated at lower-level healthcare facilities, including provincial pediatric or obstetric-paediatric hospitals (Garpvall et al., 2021). In an adult cohort from the Intensive Care–Toxicology Unit of Thai Binh Provincial General Hospital, the CRE carriage rate at admission was 41% (McConville et al., 2017). In contrast, rectal screening on admission to adult medical or surgical ICUs in the United States found a CRE colonization rate of 11% (Armin et al., 2023). At a university hospital in Thailand, the prevalence of CRE colonization at admission was 15.5% in 2022, and a cross-sectional study from Iran reported a CRE colonization prevalence of 37% among hospitalized children (van Loon et al., 2018). Taken together, our data add to the evidence that CRE carriage in healthcare facilities in low- and middle-income countries remains high and underline the urgent need for strengthened and targeted interventions.

Most confirmed CRE cases in our study (94/106; 88.7%) were detected more than 48 hours after admission, and hospitalization beyond 48 hours was associated with a 2.27-fold higher risk of CRE colonization compared with screening within 48 hours (p = 0.026). Thus, in-hospital acquisition was a major driver of CRE colonization in this setting. Length of hospital stay is a well-recognized risk factor for CRE colonization and infection, as patients are increasingly exposed to transmission via colonized or infected patients, contaminated environmental surfaces, and medical equipment (van Loon et al., 2018; Tran et al., 2019; Yen et al., 2023). The risk is amplified when hospitalization is prolonged because of severe disease or the need for invasive procedures—such as mechanical ventilation, central venous catheters, urinary catheters, or surgery—particularly in ICUs and neonatal units (van Loon et al., 2018; Tran et al., 2019; Yen et al., 2023). The high colonization pressure observed in our ICU and Neonatal Department is consistent with this pattern and reflects a combination of patient vulnerability and a challenging transmission environment.

In our study, most CRE-positive patients were admitted directly from home (travel by oneself) and were distributed across all districts and the provincial city (**Table 1**; **Appendix 1**). Our data from admission screening showed significant CRE colonization which increased during the hospital stay and which may further contribute to spread within the community after discharge. It also raises concern for increasing rates of community-onset infections—such as urinary tract caused by CRE, with consequent difficulties in selecting appropriate empirical antibiotics.

In our study, most CRE-positive patients were admitted directly from home and resided across all districts and the provincial city (**Table 1**; **Appendix 1**). High CRE prevalence was also observed among children diagnosed with sepsis at the time of the survey, and sepsis was associated with substantially higher odds of CRE colonization in multivariable analysis (adjusted OR = 6.54, 95% CI 1.55–27.64, p = 0.011). This distribution suggests that CRE colonization is not confined to a few geographic “hot spots” and raises concern both for household transmission after discharge and for spread to other healthcare facilities upon referral or readmission. It also points to a potential increase in community-onset infections, such as urinary tract caused by CRE, which may be difficult to treat empirically with standard regimens.

In this PPS, all identified CRE species showed high levels of resistance to multiple antibiotic classes (**Appendix 2**). *Escherichia coli* was the most common species (40.6%), followed by *Klebsiella pneumoniae* (34.4%) and *Enterobacter cloacae* (16.4%) (**Table 3**). The resistance profiles showed very high rates of non-susceptibility to carbapenems, extended-spectrum cephalosporins, fluoroquinolones, and β-lactam/β-lactamase inhibitor combinations (**Table 4**). CRE colonization is a recognized prerequisite to healthcare-associated infections (HAIs) caused by CRE and therefore represents a latent clinical risk: if infection occurs, therapeutic options are limited and the probability of treatment failure increases (Kramer et al., 2013; Tischendorf et al., 2016) These findings emphasize the importance of identifying colonized patients early and preventing progression to infection whenever possible.

To date, Viet Nam still lacks comprehensive national data on CRE carriage, particularly due with respect to low screening capacity and systematic sharing of data on CRE colonization status between facilities. Notification of CRE carriage at inter-facility transfer is not uniformly implemented across the country, which hampers coordinated infection prevention and control (IPC) measures. Early and accurate detection of CRE colonization is essential for identifying outbreaks and guiding IPC and treatment strategies (Zaidah et al., 2017). The cost of CRE screening depends on consumables, reagents, staff time, and local resource constraints and therefore differs between high-income and low- and middle-income countries (World Health Organization, 2019). For example, the average cost of a single CRE screening test was estimated at $8.65 in the United States (Lin et al., 2022). In contrast, in our study the material cost for one CRE screening plate was approximately $2, which could be used for two patients, thus a laboratory cost of about $1 per patient. Nonetheless, CRE screening is currently not reimbursed by health insurance and is not routinely performed in Vietnamese healthcare facilities, limiting its uptake despite its relatively low direct cost in this setting.

CRE screening and cohort care are widely recognized as key components of effective CRE control programs. At Vietnam National Children’s Hospital, admission screening of more than 900 children to three ICUs, followed by cohorting according to CRE status, reduced CRE acquisition from 90% to 48% (Garpvall et al., 2021). In Israel, a nationwide intervention combining CRE screening with cohort and isolation care significantly reduced CRE transmission (Schwaber and Carmeli, 2014). Our findings, together with these previous results, support the implementation of targeted screening strategies and cohort care in high-risk pediatric departments, particularly ICUs and neonatal units, as part of a broader IPC package.

This study has several limitations. First, it is based on a single PPS conducted in one provincial pediatric hospital, and thus not representative for Viet Nam. Nevertheless, the PPS provides a detailed snapshot of the wards studied and it may be relevant for similar provincial pediatric hospitals. Second, genotyping data are not presented here; however, a companion publication using the same isolate collection reported carbapenemase genes in 63.8% of isolates, with NDM being the predominant carbapenemase in 86.7% of CRE isolates (Pham et al., 2024). For the other 36.2% of isolates please see pham et al (Pham et al., 2024). Third, due to low reported HAI incidence and limited routine culture before starting antibiotics, we were unable to systematically link colonizing and clinical isolates and therefore could not quantify the progression from colonization to infection. Previous studies, however, have shown a strong association between CRE colonization and subsequent CRE HAI (Tischendorf et al., 2016). Finally, as a PPS, this study captures a single time point and does not account for temporal fluctuations in colonization pressure or the incidence of new acquisitions over time.

## Conclusion

In summary, we report a high prevalence of CRE colonization among pediatric inpatients in a Vietnamese provincial hospital, with evidence of substantial in-hospital transmission and a predominance of multidrug-resistant *E. coli*, *K. pneumoniae*, and *E. cloacae*. The particularly high colonization rates in ICU and neonatal wards, and among children with prolonged hospital stay or sepsis, highlight priority targets for IPC interventions, CRE screening, and cohort care. These findings form an important baseline for the ongoing randomized controlled trial on interventions to reduce CRE colonization and transmission in hospitals, households, communities, and domesticated animals, and underscore the urgent need to strengthen CRE control strategies in pediatric healthcare settings in Vietnam.

## Data Availability

The raw data supporting the conclusions of this article will be made available by the authors, without undue reservation.

## Group members of I-CRECT Consortium

^A.^ Håkan Hanberger^1,2^, Bent Jörgensen^2,3^, Åse Östholm ^1^, Lennart E Nilsson^1^
^B.^ Mattias Larsson^2,4^, Linus Olson^2,4,5^,
^C.^ Thirumalaisamy P. Velavan ^6,7^, Song H. Le^7,8^, Alexa Purgreth^6,^Mai Thi Pham^6,7^
^D.^ Flavie Goutard^9^, Etienne Loire^9^,
^E.^ Y.M. Gildas Hounmanou^10^, Anders Dalsgaard^10^,
^F.^ Phuc D. Pham^11^, Hanh T.T. Tran^12^, Nhung T. T.Nguyen^13^
^G.^ Dien M.Tran^14,16^, Phuc H. Phan^2,14^, Ngoc T.B Hoang^17^ Van Anh Thi Dinh^18^, Ngai K. Le^18^
A. Linköping University, Linkoping, Sweden
B. Karolinska Institutet, Stockholm, Sweden
C. University of Tübingen, Tübingen, Germany
D. The French Agricultural Research Centre for International Development (CIRAD), Montpellier, France
E. University of Copenhagen, Copenhagen, Denmark
F. Hanoi University of Public Health, Hanoi, Vietnam
G. Vietnam National Children’s Hospital, Hanoi, Vietnam
1. Division of Infectious Diseases, Department of Biomedical and Clinical Sciences, Faculty of Medicine, Linköping University, Linköping, Sweden,
2. Training and Research Academic Collaboration (TRAC) – Sweden – Vietnam
3. Göteborg university, Gothenburg, Sweden.,
4. Department of Global Public Health, Karolinska Institutet, Stockholm, Sweden.,
5. Department of Women’s and Children’s Health, Karolinska Institutet, Sweden.
6. Institute of Tropical Medicine, University of Tübingen, Tübingen, Germany.,
7. VGcare, Hanoi, Vietnam
8. 108 Military Central Hospital, Hanoi, Vietnam
9. Department of Vietnam, The French Agricultural Research Centre for International Development (CIRAD), Montpellier, France
10. University of Copenhagen, Copenhagen, Denmark
11. VOHUN, CERFER, Hanoi University of Public Health, Hanoi, Vietnam.,
12. Department of Research Management and Cooperation, Hanoi University of Public Health, Hanoi, Vietnam.,
13. Fundamental Sciences, Hanoi University of Public Health, Hanoi, Vietnam.,
14. Director board, Vietnam National Children’s Hospital, Hanoi, Vietnam
16. University of Medicine and Pharmacy - Vietnam National University, Hanoi, Vietnam
17. Department of Microbiology, Vietnam National Children’s Hospital, Hanoi, Vietnam
18. Department of Infection control, Vietnam National Children’s Hospital, Hanoi, Vietnam

## Appendix 1

Geographic distribution of CRE-positive inpatients and all hospital inpatients during August 2022 in a provincial pediatric hospital in the Red River Delta, Vietnam,

The dot map illustrates the home locations of all inpatients during August 2022 and, specifically, of those identified as CRE colonized on the survey date (15 August 2022). CRE-positive children were distributed across all districts of the province, indicating that colonization was not confined to a single locality or cluster. This widespread geographic distribution suggests a potential risk for post-discharge community dissemination, as colonized children may introduce CRE into household and community environments after returning home.

## Appendix 2

Colonization by different CRE species and associated multidrug-resistance patterns.

The classification of multidrug-resistant (MDR) and extensively drug-resistant (XDR) phenotypes followed the definitions proposed by Magiorakos et al (Magiorakos et al., 2012)According to these criteria, MDR is defined as acquired non-susceptibility to at least one agent in three or more antimicrobial categories, while XDR is defined as non-susceptibility to at least one agent in all but two or fewer antimicrobial categories (i.e., isolates remain susceptible to only one or two antimicrobial categories).

In this study, antibiograms for CRE isolates were constructed using eleven antimicrobial categories, including: aminoglycosides; fluoroquinolones; antipseudomonal penicillins combined with β-lactamase inhibitors; carbapenems; non-extended spectrum cephalosporins, first- and second-generation cephalosporins; cephamycins; extended-spectrum cephalosporins, third- and fourth-generation cephalosporins; penicillins; penicillins with β-lactamase inhibitors; phosphonic acids, and folate pathway inhibitors.

These categories were used to characterize the resistance profiles of *Escherichia coli*, *Klebsiella pneumoniae*, *Enterobacter cloacae*, and other CRE species isolated in this survey.

## Appendix 3

**Table:** Key characteristics of the provincial paediatric hospital in the Red River Delta, Vietnam.

Indicator	August 2022	Total 2022
Number of clinical departments	15*	15*
Number of beds	600	600
Number of healthcare workers (doctor, nurse, technician, and others)	489	505
Inpatient admission	4,053	25,620
Average duration of hospital stay (days)	7.6	7.4
Inpatient discharge	2,581	24,260
Severe cases at discharge	0	20
Deaths	3	25
Hospital-acquired cases (HAI)	6	72

(*): Thirteen clinical departments admitting inpatients were included in this study. Screening was not performed in the Outpatient Clinics or the Anaesthesia & Operating Theatre Departments, as they do not manage admitted patients.

## Glossary

AMR: Antimicrobial resistance
AST: Antimicrobial susceptibility testing
CRE: Carbapenem resistant Enterobacterales
ESBL: Extended spectrum beta-lactamase
HAI: Hospital acquired infection
I-CRECT: Interventions to decrease CRE colonization and transmission
ICU: Intensive care unit
IPC: Infection prevention and control
JPIAMR: Joint programming initiative on antimicrobial resistance
MDR: Multidrug-resistant
NICU: Neonatal intensive care unit
PPS: Point prevalence survey
PPH: Provincial pediatric hospital
WHO: World Health Organization
XDR: Extensively drug-resistant
VNCH: Vietnam National Children’s Hospital
